# Variations of OH defects and chemical impurities in natural quartz within igneous bodies

**DOI:** 10.1007/s00269-020-01091-w

**Published:** 2020-05-05

**Authors:** Alexander Potrafke, Karel Breiter, Thomas Ludwig, Rolf Dieter Neuser, Roland Stalder

**Affiliations:** 1 Institut für Mineralogie und Petrographie, Universität Innsbruck, Innrain 52f, 6020 Innsbruck, Austria; 2 Institute of Geology, Czech Academy of Sciences, Rozvojová 269, 16500 Praha 6, Lysolaje Czech Republic; 3 Institut für Geowissenschaften, Universität Heidelberg, Im Neuenheimer Feld 234-236, 69120 Heidelberg, Germany; 4 Institut für Geologie, Mineralogie und Geophysik, Ruhr-Universität Bochum, Universitätsstraße 150, 44801 Bochum, Germany

**Keywords:** Quartz, OH defects, IR spectroscopy, SIMS, Cathodoluminescence, Granite

## Abstract

In this study, we present the first systematic dataset on natural variations of OH defect and trace element contents in quartz within igneous bodies. Samples were derived from bore holes of two plutonic bodies from the Krušné Hory/Erzgebirge (German–Czech border), representing typical A-type (Cínovec/Zinnwald granite cupola) and S-type (Podlesí Stock) granite intrusions. Fourier Transform Infrared spectroscopy of quartz was used to investigate the sample set with regard to its OH defect speciation and content. For Zinnwald quartz, IR absorption spectra reveal different lithologies due to changes of the OH defect inventory, enabling a subdivision of the granitic body: (1) hydrothermal greisen quartz of the uppermost part of the intrusion have low OH defect contents (average of 15 µg/g H_2_O); (2) zinnwaldite granite quartz vary strongly in defect content and show the highest content of the dataset (10–70 µg/g H_2_O); (3) quartz from an underlying biotite granite have slightly lower, but very uniform contents down to the bottom of the borehole at 1600 m (average 20 µg/g H_2_O). Infrared spectra of Podlesí quartz reveal a gradual increase in total defect water content with increasing depth over 350 m (30–55 µg/g H_2_O). Lithium contents in quartz samples from the uppermost part of the Zinnwald intrusion correlate with the occurrence of Li-specific OH defects, while cathodoluminescence (CL) images do not show specific differences. Our findings evidence the potential of OH defects in quartz as a tool to decipher differentiation trends in igneous bodies, and the application of their eroded material for provenance analyses.

**Electronic supplementary material:**

The online version of this article (10.1007/s00269-020-01091-w) contains supplementary material, which is available to authorized users.

## Introduction

Quartz (SiO_2_) is a major constituent of felsic igneous rocks such as granites, as well as of metamorphic and sedimentary rocks. In unconsolidated sedimentary material, quartz usually makes up the principal component due to its high resistance to chemical and mechanical weathering. Although quartz does not form solid solution series, it is capable of incorporating significant amounts of trace elements such as P^5+^, Ti^4+^, Ge^4+^, Al^3+^, Fe^3+^, B^3+^, Li^+^, Na^+^, K^+^ and H^+^ (Bambauer 1961, 1963; Kats 1962; Aines and Rossman 1984; Müller and Koch-Müller 2009). Cations can be incorporated either by exchange of cations isovalent to Si^4+^ (e.g. Ti^4+^, Ge^4+^) or via coupled substitutions (e.g. Si^4+^ = Al^3+^ + H^+^). Participation of hydrogen in the substitution mechanism leads to the formation of hydroxyl dipoles (OH) with the oxygen anions from the quartz lattice. The total concentration is commonly expressed as neutral water component (in the following as µg/g H_2_O or “defect water” content), which is geochemically appropriate since oxygen is the only relevant anion. Molecular water from fluid inclusions was ignored in this study as its concentration is not controlled by thermodynamics. In contrast, trace element incorporation in general and OH defect formation in particular is a function of physico-chemical parameter such as pressure, temperature, chemical composition and water fugacity. These parameters may change during the genesis of a magmatic body, either abruptly (due to multiple magma injections) or over time (during solidification and potential post-genetic overprint), leaving behind igneous bodies with complex genetic histories. Especially the availability of water plays a crucial role by affecting the amount of the generated granitic melt, its mobility, crystallisation sequences and temperatures (Tuttle and Bowen 1958; Holtz and Johannes 1994).

Consequently, similar to the successful application of trace metal incorporation in quartz as geothermometer (Wark and Watson 2006; Huang and Audétat 2012), OH defects in quartz provide detailed information on the prevailing conditions in granitic melts and thus can be applied as tracer for crystallisation conditions of the parental igneous body.

**Fig. 1:**
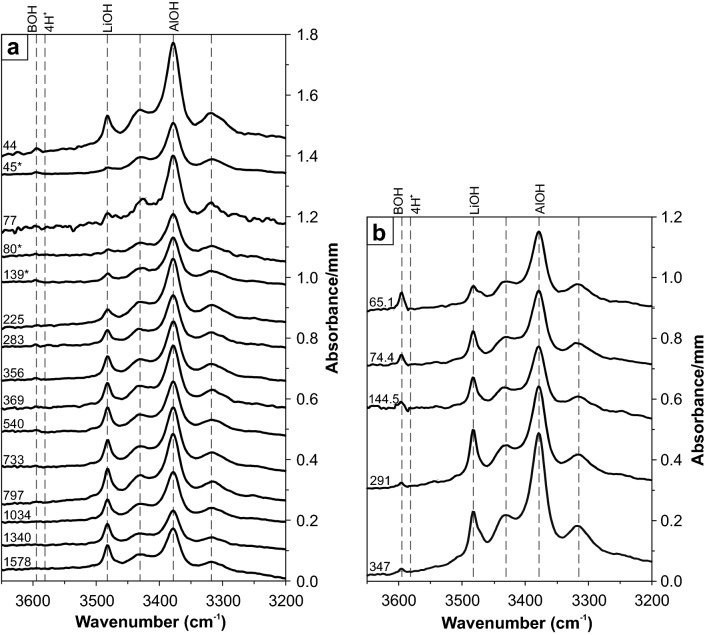
IR spectra of quartz in the OH spectral region (*E*||*n*_o_–*E*||*n*_e_), averaged for all samples of each of each drill hole depth, from **a** Zinnwald borehole CS-1 and **b** Podlesí boreholes PTP-1 and PTP-3. Spectra are normalised to 1 mm thickness and offset vertically for clarity. Numbers on the left indicate sample depths measured from upper borehole surface as given in Table 1, and asterisks indicate samples from greisen. BOH, LiOH, AlOH and 4H^+^ refer to B-, Li-, and Al-specific OH defects as well as hydrogarnet substitution, respectively

To investigate hydrous defects in nominally anhydrous minerals qualitatively (defect species) and quantitatively (defect water content), Fourier Transform Infrared (FTIR)-spectroscopy is a powerful method. OH dipoles give rise to absorption bands in the IR spectra at specific wavenumbers (Kats 1962; Bambauer 1963; Aines and Rossman 1984; Rovetta 1989; Thomas et al. 2009; Stalder and Konzett 2012; Baron et al. 2015) and can be assigned to particular hydrous defects (Fig. 1). Thus, the presence of different defect species and respective absorption band heights can be used to distinguish between different populations or generations within igneous bodies.

However, the characterisation of large populations of quartz grains from natural reservoirs by FTIR spectroscopy is rather new. Earlier and more frequently used methods are based on the emission colour by cathodoluminescence and the trace element content.

Cathodoluminescence (CL) microscopy/spectroscopy is a fast and powerful tool to distinguish between quartz of volcanic, plutonic, high-grade to low-grade metamorphic or authigenic origin, and to reveal chemical zoning (Zinkernagel 1978; Müller et al. 2000; Augustsson and Reker 2012). Visible luminescence colours in CL microscopy depend on the relative intensities of the dominant emission bands between 380 and 700 nm generated by metal impurities (Götze et al. 2001). The usually very broad blue luminescence band consists of overlapping component bands, which can be related to different types of centres. Some of the CL emissions of quartz from this spectral region (175 nm, 290 nm, 340 nm, 420 nm, 450 nm, 580 nm) can be related to intrinsic lattice defects (Stevens-Kalceff and Phillips 1995; Gorton et al. 1997). Extrinsic defects such as the alkali (or hydrogen)-compensated [AlO_4_/M^+^] centre have been suggested as being responsible for the transient emission band at 380–390 nm (Luff and Townsend 1990) and the short-lived blue-green CL centred around 500 nm (Ramseyer and Mullis 1990; Perny et al. 1992). CL emissions between 620 and 650 nm in the orange to red spectral region are attributed to the non-bridging oxygen hole centre (NBOHC) with several precursors (Stevens-Kalceff 2009).

In addition to CL, quartz has been analysed for its trace element content (Watt et al. 1997; Müller et al. 2003a, b; Larsen et al. 2004; Ackerson et al. 2015, Kleine et al. 2018), some of them allowing application as geothermometers (Wark and Watson 2006; Huang and Audétat 2012).

Although OH defects in quartz have been proposed as a tool for quartz provenance to distinguish sedimentary, igneous and metamorphic lithologies (Stalder and Neuser 2013; Stalder 2014; Stalder et al. 2017, 2019), literature still lacks detailed studies on the hydrous defect inventory of quartz from individual granitic bodies. In this study, we present the first systematic dataset on OH defects in quartz from borehole sections of two localities to monitor variations in selected granitic bodies. For this purpose, a combination of the above-described methods (FTIR spectroscopy, SIMS, CL spectroscopy) was applied to the same set of crystals. Our findings will be evaluated with respect to the incorporation mechanism of impurities in quartz, the zonation in plutonic bodies and the potential of quartz as tool for provenance analyses of sedimentary material.

## Geological setting and samples

The late-Variscan granites and associated acid volcanic rocks of the Krušné Hory/Erzgebirge (German–Czech border) form a ca. 80-km-long NE–SW-oriented belt intruded into the Variscan crystalline basement of the Saxo-Thuringian domain in the NW part of the Bohemian Massif (Hoth et al. 1995; Cháb et al. 2010; Linnemann and Romer 2010) in a relatively short period from ~ 330 to 310 Ma (Förster and Romer 2010; Ackerman et al. 2017). Two types of magma were generated and emplaced synchronously during this event (Breiter 2012): (1) strongly peraluminous P-rich (S-type) melts dominated in the western and central part of the Erzgebirge and (2) slightly peraluminous P-poor melts (A-type), forming both intrusive and volcanic rocks mainly in the eastern part of the area. Both magmatic suites culminated with strongly fractionated subvolcanic granite intrusions followed by Sn + W ± Li–Nb–Ta mineralisation of the greisen type. The shallow level of the granite intrusions is indicated by the identification of pipes of explosive breccia at the top of several hidden or only partially exposed intrusions (Seltmann and Schilka 1995).

**Fig. 2:**
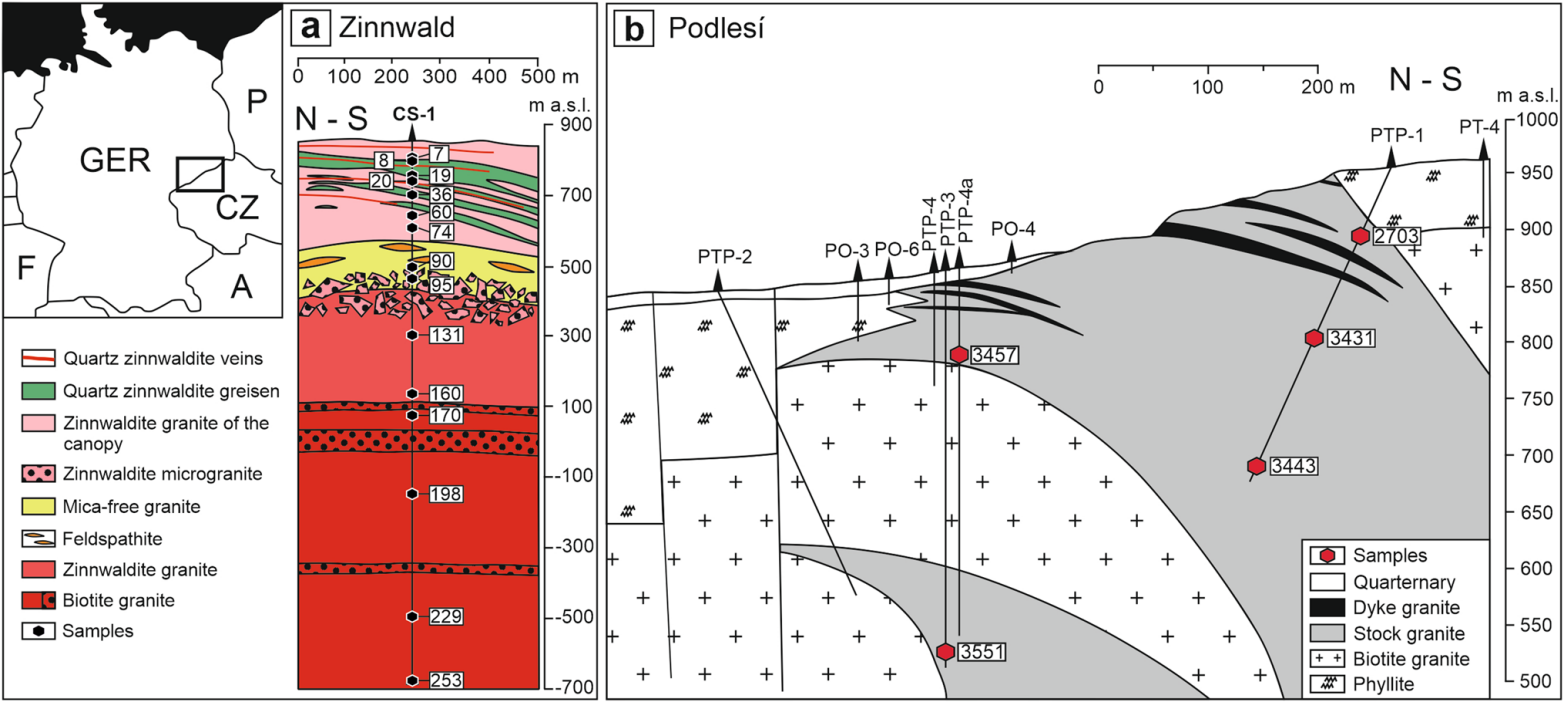
Sections of **a** borehole CS-1 penetrating the Zinnwald/Cínovec granite cupola and **b** Podlesí plutonic body with the positions of existing drill holes. Hexagons mark samples investigated in this study. *GER* Germany, *CZ* Czech Republic, *P* Poland, *F* France, *A* Austria

For our study, we selected two geologically well-categorised intrusions (Fig. 2) that are typical for the subaluminous (A-type, Zinnwald/Cínovec) and strongly peraluminous (S-type, Podlesí) granites. Samples were taken from deep boreholes, enabling the study of quartz in intervals along vertical sections of 1600 m (Zinnwald/Cínovec) and 350 m (Podlesí), respectively.

### Zinnwald/Cínovec

The Zinnwald/Cínovec granite lies on both sides of the Czech–German border, forming a ca. 20-km-long NW–SE-oriented belt. The pluton represents strongly fractionated subaluminous to slightly peraluminous A-type granites (Breiter et al. 2017b) and consists of two main types of granite (Štemprok and Šulcek 1969): (1) the older medium- to fine-grained porphyritic biotite granites compose the majority of the known volume of the pluton; (2) the younger medium- to fine-grained granular albite–topaz–zinnwaldite granites accompanied by Sn–W (Nb, Ta, Mo, Sc) mineralisation of greisen and vein types form small, mostly hidden intrusions. Albite–topaz–zinnwaldite granite (ZiG) was found in several textural varieties to a depth of 735 m, while albite–Li–biotite granite (BtG) was found at greater depths (Fig. 2a). A fractionation in situ can be observed in zinnwaldite granite by increasing concentrations of volatile and lithophile elements upward.

Contents of Al, Ti, Li as well as Al/Ti and Ge/Ti ratios in quartz reflect the degree of fractionation of the parental melt from which primary quartz crystallised (Breiter et al. 2017a). From the BtG to the younger ZiG, quartz is characterised by increasing contents of Al (from 136–176 to 240–280 µg/g) and decreasing Ti (from 16–54 to 6–14 µg/g), while the contents of Li and Ge are similar (15–36 and 0.8–1.7 µg/g, respectively). Quartz from greisen is poor in all measured elements (26–59 µg/g Al, 0.5–1.6 µg/g Ti, 2–13 µg/g Li, 0.8–1.6 µg/g Ge). Within the greisen, remnants of primary magmatic quartz can be distinguished from metasomatic greisen-stage quartz in their higher intensity of CL and relatively higher Ti contents.

Samples for this study are derived from the vertical section through Zinnwald/Cínovec granite. 15 drill core samples from different depths and lithologies of bore hole CS-1 (Štemprok and Šulcek 1969) were selected.

### Podlesí

The Podlesí granite crops out in the western part of the Erzgebirge and is composed of relatively older barren biotite granites followed by F-enriched Sn-bearing albite–topaz–biotite granites. The most fractionated residual melts of these magmas gave rise to small intrusions of extremely F-, P- and Li-rich granites making up the Podlesí stock (Fig. 2b, Breiter et al. 2005). The Podlesí granite suite forms a tongue-like intrusion of albite–topaz–protolithionite granite (stock granite) with the upper contact rimmed by a marginal pegmatite. At a depth of 40–100 m, the stock granite is intercalated with flat dykes of albite–topaz–zinnwaldite granite (dyke granite). Prominent layering and unidirectional solidification textures indicate extreme enrichment of fluxing elements and undercooling of the crystallised melt. Metasomatic greisens are subordinate.

Six quartz populations (PQ1–6) from different intrusion stages were distinguished in Podlesí (Breiter and Müller 2009). From an early magmatic quartz in the stock granite (PQ1) to oriented comb quartz layers in a late dyke granite (PQ6) contents of Al and Li increased by factors of 3: from 400 to 1000 µg/g Al and 33 to 96 µg/g Li, while Ti decreased from 60 to 13 µg/g.

For this study, five drill core samples from two different boreholes and varying depths of the intrusion have been chosen for further preparation. To document vertical variations in the same quartz generations, sample selection was limited to the albite–topaz–protolithionite granite (stock granite). Thus, only generations PQ1 and PQ2 from the first intrusion stage are compared.

## Methods

### FTIR spectroscopy and OH defect quantification

For each sample five oriented quartz wafers were prepared for IR spectroscopy following the protocol described by Stalder and Konzett (2012), adding up to a total of 75 (Zinnwald) and 25 (Podlesí) crystals (Table 1). Granite samples were manually crushed with a hammer, and the 0.5–1 mm fraction was extracted by sieving. Individual quartz crystals were handpicked randomly (regardless of any preferential characteristics) and oriented parallel to the c-axis in thermoplastic resin on a glass slide. Crystal alignment was checked with an optical microscope. Successful orientation was confirmed by birefringence values of Δ*n* = 0.009 in orthoscopic and “flash figures” in conoscopic illumination. Oriented crystals were manually ground and polished on both sides, and the remaining resin was removed by rinsing in acetone. The crystal thickness, a crucial parameter for water quantification, was determined by a mechanical Mitutoyo micrometer gauge with an accuracy of ± 2 μm, and crosschecked by the absorption band of the lattice overtone at 1793 cm^−1^ (Stalder et al. 2017). Crystal thicknesses in the investigated sample set range from 80 to 250 µm.

**Table 1:** Granite samples from Zinnwald borehole CS-1 and Podlesí boreholes PTP-1 and PTP-3 with the average defect water content of analysed quartz crystals

Sample #	Petrological classification	Borehole depth (m)	Total absorbance^a^	OH defect content (µg/g H_2_O)			
				T (09)		L&R (97)	
				Range	Average	Range	Average
	**Zinnwald**						
7	Finegrained Zinnwaldite Granite of the canopy	44	24.7	18.2–54.7	38.8	18.4–71.6	38.8
8	Greisen	45	11.2	16.5–18.7	17.2	15.8–18.6	17.2
19	Finegrained Zinnwaldite Granite of the canopy	77	15.9	16.7–32.5	24.3	16.4–33.0	24.8
20	Greisen	80	10.6	8.6–27.9	16.2	8.7–27.5	16.4
36	Greisen	139	9.8	13.0–18.7	14.9	13.1–19.3	15.3
60	Zinnwaldite Granite of the canopy	225	13.9	10.4–37.7	21.2	10.9–38.8	21.7
74	Zinnwaldite Granite of the canopy	283	12.2	9.4–28.5	18.6	9.5–28.5	19.1
90	Mica-free Granite	356	14.5	14.4–27.9	22.1	14.6–29.5	22.9
95	Micro Granite	369	16.0	30.1–11.1	24.4	11.1–30.8	25.2
131	Zinnwaldite Granite	540	13.6	14.2–24.1	20.8	15.0–25.9	21.2
160	Zinnwaldite Granite	733	15.8	22.4–27.3	24.1	23.6–27.8	25.0
170	Bt-Granite	797	17.4	24.0–31.0	26.5	25.1–31.3	27.2
198	Bt-Granite	1034	12.7	12.3–22.4	19.3	12.6–23.0	20.0
229	Bt-Granite	1340	9.1	10.9–18.3	13.9	11.2–19.2	14.5
253	Bt-Granite	1578	12.2	15.3–21.8	18.6	16.3–22.7	19.6
	**Podlesí**						
2703	Stock Granite^b^	65.1	18.3	23.9–30.8	28.0	25.9–32.2	29.8
3457	Stock Granite^c^	74.4	19.9	15.5–40.0	30.2	17.8–41.4	31.8
3431	Stock Granite^b^	144.5	17.9	21.2–32.0	27.9	22.2–33.4	29.2
3443	Stock Granite^b^	291.0	25.2	27.5–50.4	38.4	29.4–53.0	40.5
3511	Stock Granite^c^	347.0	33.4	42.3–65.1	51.0	44.0–67.7	53.2

T (09) = calculated using the calibration of Thomas et al. (2009)

L&R (97) = calculated using the calibration of Libowitzky and Rossman (1997)

^a^Average integrated total absorbance in the range 3250–3600 cm^−1^

^b^Borehole PTP-1

^c^Borehole PTP-3

In contrast to most other methods, polarised IR-spectroscopy measurements allow to distinguish between molecular water (H_2_O), causing a broad band between 3000 and 3700 cm^−1^, and water from OH defects, causing relatively sharp absorption bands at characteristic wavenumbers between 3250 and 3600 cm^−1^ (Kats 1962). While in quartz most OH defect absorption bands are perfectly polarised perpendicular to the optical axis (*E*||*n*_o_), the molecular water signal exhibits isotropic behaviour (identical absorbance in all crystallographic directions). Thus, the polarised measurement *E*||*n*_o_ reveals the OH defects plus molecular water, *E*||*n*_e_ reveals the molecular water only, and the contribution of the OH defects is derived by the subtraction *E*||*n*_o_ − *E*||*n*_e_. The only deviation from this protocol is the B-related OH absorption band at 3595 cm^−1^ that is not perfectly aligned perpendicular to the optical axis and shows a small contribution for *E*||*n*_e_ (Baron et al. 2015). For crystals that showed the BOH absorption band, the respective range (3585–3605 cm^−1^) in the IR spectrum was corrected by adding 1.5 times the *E*||*n*_e_ component to the *E*||*n*_o_ − *E*||*n*_e_ difference, resulting in *E*||*n*_o_ + 0.5 *E*||*n*_e_ for this region.

Infrared spectra were recorded at room temperature in transmission mode by using a Bruker Vertex 70 FTIR spectrometer coupled to a Hyperion 3000 microscope equipped with a 15 × Cassegrain objective with a numerical aperture of 0.4, nitrogen-cooled MCTD316-025 (mercury cadmium telluride) detector, a SiC globar light source, a KBr beamsplitter and a wire ZnSe grid polariser. The beam path was continuously flushed with dry air and samples were placed on a BaF_2_ plate. 32–500 scans were conducted on background and sample with a resolution of 4 cm^−1^ and aperture sizes between 40 × 40 and 150 µm × 150 μm in the wavenumber range of 7000–550 cm^−1^. On each crystal two to four visually inclusion-free volumes were analysed with two measurements each (one *E*||*n*_o_, one *E*||*n*_e_), by turning the polariser for 90° after the first measurement. The two polarised IR spectra were subtracted (*E*||*n*_o_ − *E*||*n*_e_), normalised to thickness and baseline-corrected by a linear baseline between 3600 and 3250 cm^−1^. Defect water contents (Table 1) were calculated by integration in the spectral region (3600–3250 cm^−1^), using the integrated extinction coefficients from mineral-specific (Thomas et al. 2009) and general wavelength-specific (Libowitzky and Rossman 1997) calibrations. Results are expressed as µg/g H_2_O, which is equivalent to the notation ppm weight H_2_O. The analytical error for water quantification is estimated as 10%. Error sources are (1) mismatch in crystal orientation, (2) uncertainties in thickness determination, and (3) uncertainty in baseline correction.

### IR imaging

In order to visualise possible chemical heterogeneities, the OH distribution of selected crystals was monitored using the focal plane array (FPA) detector. The FPA consists of 64 × 64 MCTD364 detectors, resulting in 4096 simultaneously recorded spectra in the range of 4000–900 cm^−1^ on an area of 170 µm × 170 µm with a pixel resolution of 2.7 µm. This implies that the spatial resolution of the MIR signal (wavelength divided by the double numerical aperture) is limited by the IR radiation and not by the pixel resolution (e.g. at wavelengths of 3 µm, corresponding to 3300 cm^−1^, and a numerical aperture of 0.4 the optical resolution is 3.75 µm). Larger areas can be imaged by sequential analysis of several IR images. All spectra were automatically integrated in different spectral regions of interest, thus enabling a quick overview over the OH absorption bands and the absorptions caused by the lattice overtones (calculating the thickness by the method of Stalder et al. 2017). The ratio of both is used for automatic thickness normalisation.

### Cathodoluminescence (CL)

From the quartz wafer set that has been investigated by IR spectroscopy (Table 1), two crystals per sample depth were selected for CL spectroscopy, adding up to 40 wafers in total (30 for Zinnwald, 10 for Podlesí). Crystals were mounted on rectangular thin section slides and polished to a thickness of 80 μm. Additionally, seven thin sections (4 for Zinnwald, 3 for Podlesí) from the granite hand-specimen were prepared for a general overview. After applying a thin carbon coating (< 3 nm), the samples were studied using a hot-cathode CL device at 14 kV beam energy (HC1-LM, LUMIC; Neuser et al. 1996). Photos were taken by a highly sensitive high-resolution digital microscope camera (DP73, Olympus). The CL microscope is connected to a triple grating spectrograph (Model 275, Acton Research) by a quartz light guide. An ultra-sensitive Peltier-cooled CCD detector camera (PIXIS, Princeton Instruments) collects the light at the exit of the spectrograph and spectra were then processed using the WinSpec software (Princeton Instruments). Two CL spectra per crystal were recorded at the very beginning of excitation and again after 2 min, respectively, to document the incident spectral characteristics and their change over time due to the exposure to the electron beam. In addition, CL microscopy images of each quartz wafer were recorded after irradiation to document internal zonings. The application of CL microscopy allows an easy discrimination of quartz from other mineral phases in the analysed granites (e.g. plagioclase, K-feldspar, accessories).

### Secondary ion mass spectrometry (SIMS)

To permit a reliable comparison of the methods, SIMS measurements were conducted on the identical set of 25 Zinnwald quartz crystals (5 got lost during preparation) that were investigated by CL and IR before. Concentrations of Li, B, Na, Al, K, Ti and Ge were measured using a CAMECA ims3f ion microprobe at the Institute of Earth Sciences, Heidelberg University. ^16^O^−^ primary ions with a net energy of 17 keV and a beam current of ~ 15 nA were focused on an area of 15 µm in diameter on the quartz grains. Positive secondary ions were accelerated to 4.5 keV and the energy acceptance of the double-focussing mass spectrometer was set to 75 ± 20 eV (energy filtering). The mass resolving power *M*/Δ*M* was ~ 2000 (10%) and the secondary ions were detected by an electron multiplier in counting mode. To reduce the influence of surface contamination, the area analysed was limited to a diameter of ~ 12 µm using a 750 µm field aperture and a nominal imaged field of 25 µm (Marschall and Ludwig 2004). Li, B, Na, Al, K, Ti and Ge concentrations in quartz were calculated using the ion yield relative to Si (RIY) which was determined using the NIST SRM610 glass (concentrations taken from Jochum et al. 2011). The energy filtering method minimises the matrix effect (potentially inaccurate results because of chemical and/or structural differences between the reference material used for calibration and the unknown samples) and reduces the intensity of molecular interferences (e.g. Shimizu 1986; Zinner 1986; Bottazzi et al. 1992; MacRae et al. 1993; Ottolini et al. 1993, 2002; Kaliwoda et al. 2011). The repeatability (1rsd) of the calibration was < 1% for all elements except for Ge with 1.6% (see electronic appendix). The most critical isotope for molecular interference in this setup was ^74^Ge with the following interferences: ^58^Fe^16^O, ^28^Si^30^Si^16^O and ^29^Si_2_^16^O. The FeO interference was negligible in the SRM610 glass and in the quartz samples, while the contribution of the ^28^Si^30^Si^16^O and ^29^Si_2_^16^O interference on the ^74^Ge peak was reduced to ≤ 0.1% of its intensity by mass resolving power (*M*/Δ*M* ~ 1500 at 0.1%). The elimination of the ^28^Si^30^Si^16^O and ^29^Si_2_^16^O interference on ^74^Ge was demonstrated on the Herasil 102 silica glass with a measured apparent Ge concentration of 0.001 ± 0.221 µg/g (electronic appendix). All SIMS data were corrected for the instrument’s separately determined (“well-known”) background count rate of 0.025 counts/s. Based on this background, the detection limits (*x*_D_ and *x*_C_, see Currie 1968; Marschall and Ludwig 2004) were < 0.1 µg/g for all elements except for Ge with *x*_C_ = 0.25 µg/g and *x*_D_ = 0.73 µg/g (see electronic appendix for complete detection limit data). SIMS measurements were conducted on two to seven locations on each crystal depending on the crystal size. Data points with elevated concentrations of K and Al (K > 100 µg/g, Al > 1000 µg/g) were considered as contaminated by micro- and nano-inclusions (fluid, melt or mineral) and hence rejected.

## Results

### OH defect content, speciation and spatial distribution

In general, the Al-specific OH triplet at 3310, 3378 and 3430 cm^−1^ is the dominant absorption feature for both Zinnwald and Podlesí quartz crystals (Fig. 1), followed by the Li-specific absorption band at 3470–3480 cm^−1^. The BOH defect at 3595 cm^−1^ is usually subordinate, while the hydrogarnet defect (Si^4+^ = 4H^+^) at 3585 cm^−1^ is absent throughout all measurements.

Absorption spectra of Zinnwald quartz crystals (averaged over all measurements of each sample depth) show highest diversity in OH absorption band heights in the shallowest part of the body (Fig. 1a). While crystals from the granitic samples show strong contributions from both Al- and Li-specific OH defects to the absorption spectra, crystals from greisen samples show comparably low total absorbances, and the LiOH absorption band is nearly absent. Towards 735 m depth, IR spectra exhibit a steady increase in the Li-specific OH absorption band, while the band triplet assigned to Al stays more or less constant. From 735 m downwards, IR spectra show an abrupt decrease in the AlOH absorption triplet and slightly decreased LiOH band contribution.

**Fig. 3:**
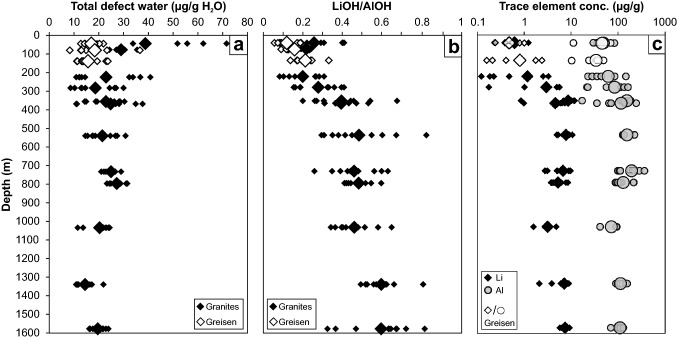
**a** Total defect water content, **b** corresponding LiOH/AlOH ratio (derived from the *I*_3480_/*I*_3378_ band ratio), and **c** Li and Al concentrations of Zinnwald quartz versus borehole depth. Larger symbols display average values for each sample depth

**Fig. 4:**
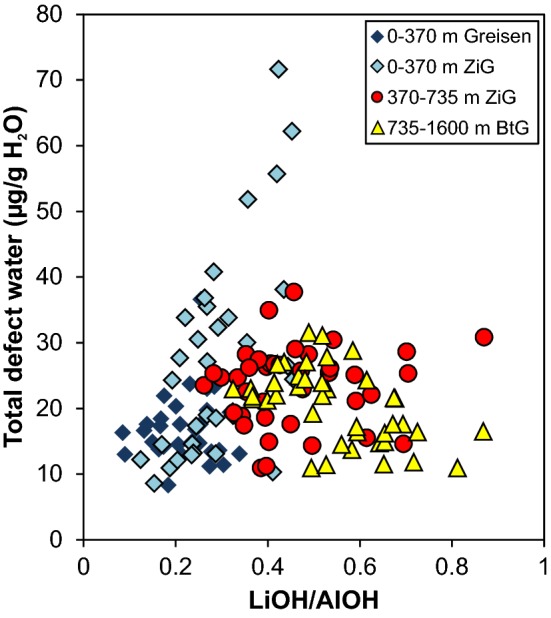
Total defect water content versus LiOH/AlOH (derived from the I_3480_/I_3378_ band ratio) for Zinnwald quartz crystals. Values are grouped to display changes between different depth levels of the granitic body

In terms of total defect water content, quartz crystals from greisen cluster in a more narrow range (10–25 µg/g) with an average of 17 µg/g, while granitic quartz crystals show a larger scatter (25 and 70 µg/g) in the uppermost part of the body (Fig. 3a). The scatter in total defect water content decreases with increasing depth towards 735 m with average values around 20 and 30 µg/g. The pronounced drop in AlOH band height from 735 m downwards is reflected by a drop of total OH defect content to values between 15 and 20 µg/g. The continuous increase in LiOH/AlOH (*I*_3480_/*I*_3378_) band ratio illustrates the steady increase in LiOH contribution up to 735 m depth, as well as the decrease in AlOH band triplet height towards higher depth (Fig. 3b). For even better visualisation, the total defect water content can be linked to the LiOH/AlOH (*I*_3480_/*I*_3378_) band ratio in a discrimination plot (Fig. 4). Transitions or abrupt changes in the OH defect inventory allow to group data to different borehole levels. Greisen samples between surface and 370 m depth cluster narrowly at low total defect water contents and low band ratios, while quartz from granite samples of the same depth show a larger scatter as well as the highest total defect water content found in the whole sample set (72 µg/g H_2_O). The granite quartz in the intermediate depth range from 370 to 735 m exhibit lower total defect water contents compared to their above counterpart, but a pronounced increase in band ratio. This trend is continued from 735 to 1600 m, where the total defect water content drops further and LiOH/AlOH increases.

**Fig. 5:**
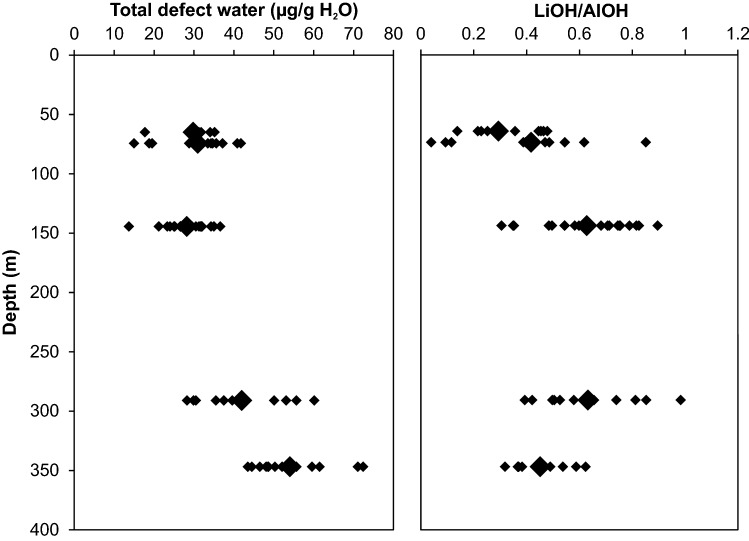
**a** Total defect water content and **b** corresponding LiOH/AlOH ratio (derived from the *I*_3480_/*I*_3378_ band ratio) versus borehole depth for Podlesí quartz crystals. Large symbols display average values for each sample depth

**Fig. 6:**
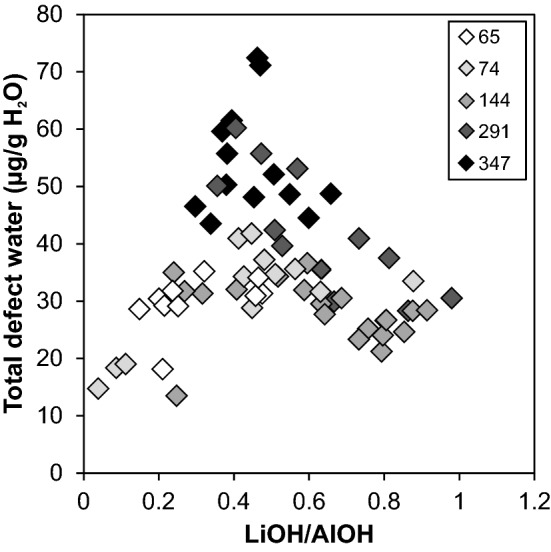
Total defect water content versus LiOH/AlOH ratio (derived from the *I*_3480_/*I*_3378_ band ratio) for Podlesí quartz crystals. Sample depths are indicated in grey shade from white (shallow) to black (deep)

Average absorption spectra from Podlesí quartz crystals reveal an increase of both AlOH and LiOH with increasing depths (Fig. 1b). The LiOH absorption band, however, is small compared to that of AlOH. A small contribution from the BOH band is present at shallow levels, but that band decreases with increasing depths and becomes negligible towards the bottom of the sampled borehole. Total defect water contents are mainly controlled by the predominant AlOH band, and thus a positive trend in total defect water content with increasing depth can be documented, rising from 30 µg/g at shallow levels to 55 µg/g at 350 m depth (Fig. 5a). This is also reflected by a decrease of LiOH/AlOH (i.e. *I*_3480_/*I*_3378_) band ratio between 290 and 350 m depth (Fig. 5b), controlled by an increase of the AlOH band height by a factor of two. Analogously to the treatment of Zinnwald quartz, crystals from Podlesí can be further discriminated by the comparison of total defect water content versus band ratio, with regard to sample depth (Fig. 6). Crystals from the upper part of the sampled borehole exhibit medium to low defect water contents combined with a wide range in band ratios. In contrast, samples from the deeper part cluster at high total defect water contents (40–72 µg/g H_2_O) with intermediate band ratios (0.3–0.7) as a result of the dominance of the AlOH defect.

**Fig. 7:**
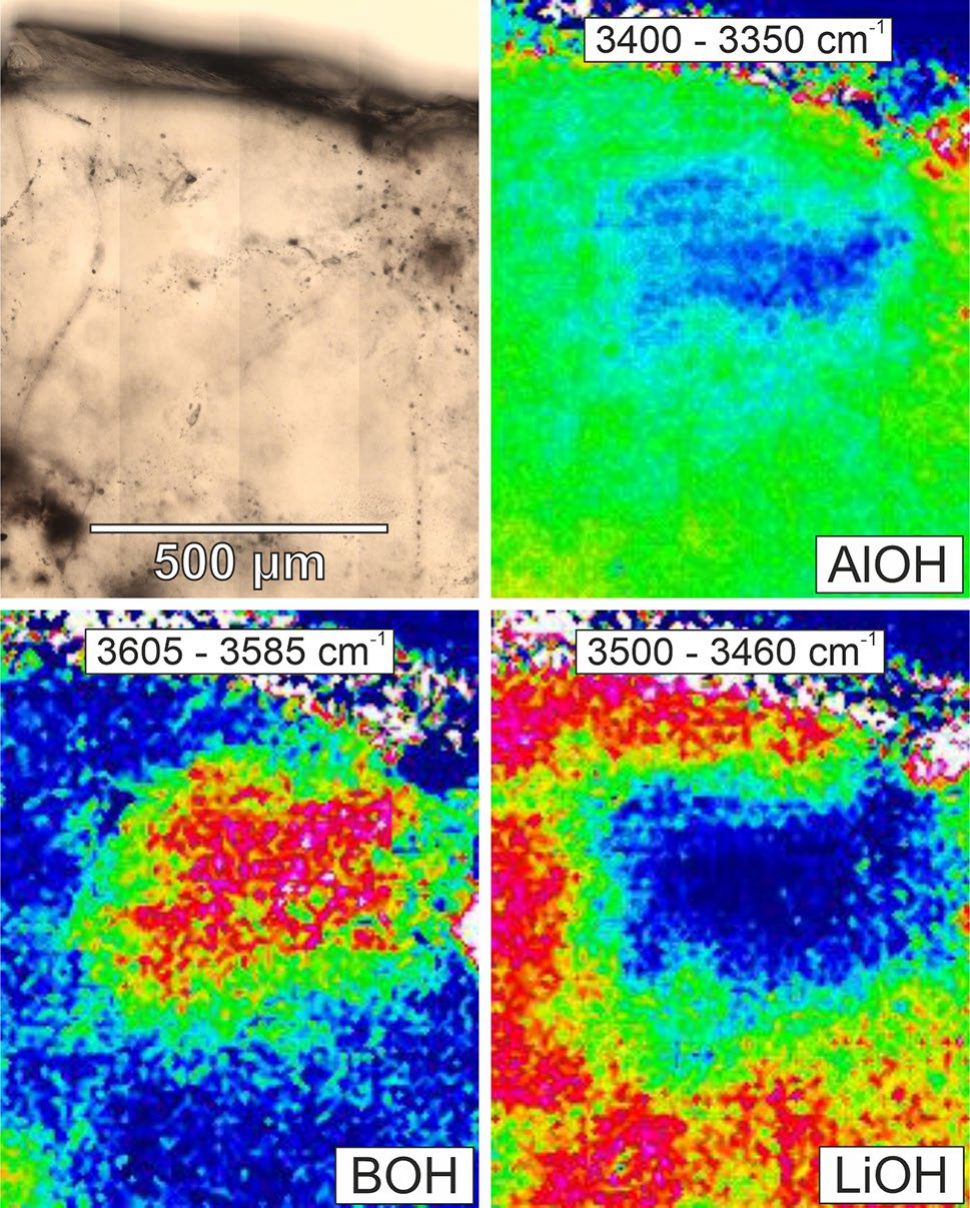
FPA image of specific OH defects in a quartz crystal from Podlesí Stock. Integrations over certain wavenumber ranges (as indicated) reveal the spatial distribution of the specific defect species (*BOH* 3605–3585 cm^−1^, *LiOH* 3500–3460 cm^−1^, *AlOH* 3400–3350 cm^−1^). Warmer colours represent higher values. Microscopy image (top left) for orientation

IR imaging occasionally revealed chemical heterogeneity of the OH defect inventory within one single crystal. Specifically, domains characterised by B-, Li-, and AlOH defect distribution differ significantly from the remaining part of the crystal (Fig. 7), indicating several stages of formation conditions. This feature was only observed in one crystal, but may appear elsewhere in the investigated section.

### Cathodoluminescence (CL)

**Fig. 8:**
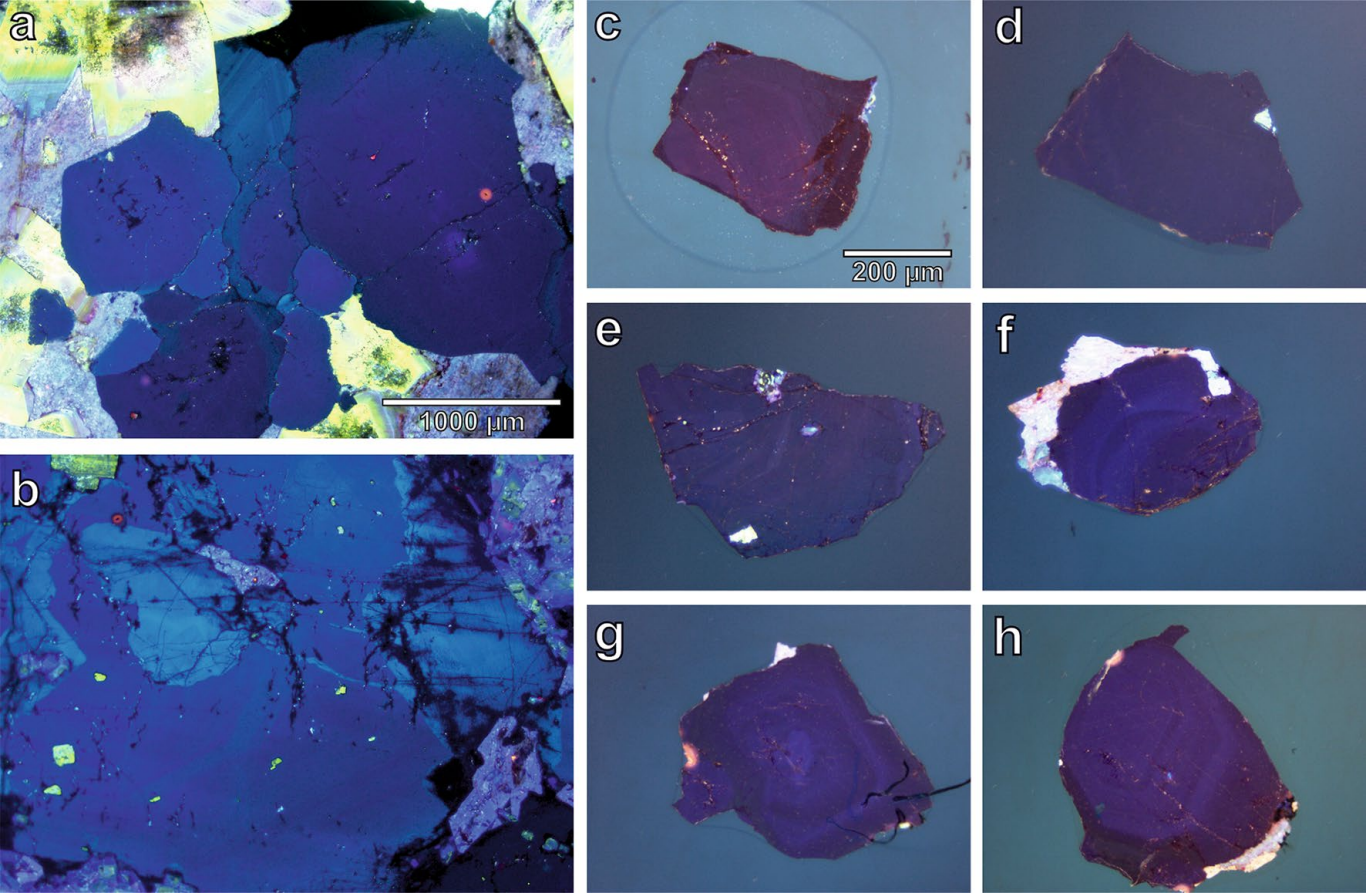
CL microscopy images of two thin sections (**a**, **b**) and six single crystals (**c**–**h**) from Zinnwald as examples for crystal zoning in the investigated sample set. **a** Sample #90, 356 m depth, **b** sample #253, 1578 m depth, **c** quartz AP119, 225 m depths, **d** quartz AP076, 540 m depth, **e** quartz AP077, 733 m depth, **f** quartz AP136, 797 m depth (bright blue is feldspar), **g** quartz AP145, 1340 m depth, **h** quartz AP087, 1578 m depth

Thin section overviews exhibit quartz with typical light to deep blue colours and reveal internal zonings (Fig. 8a, b). Likewise, the internal structure of some of the quartz grains analysed by FTIR is exposed, showing weak to absent zoning (Fig. 8c–h).

**Fig. 9:**
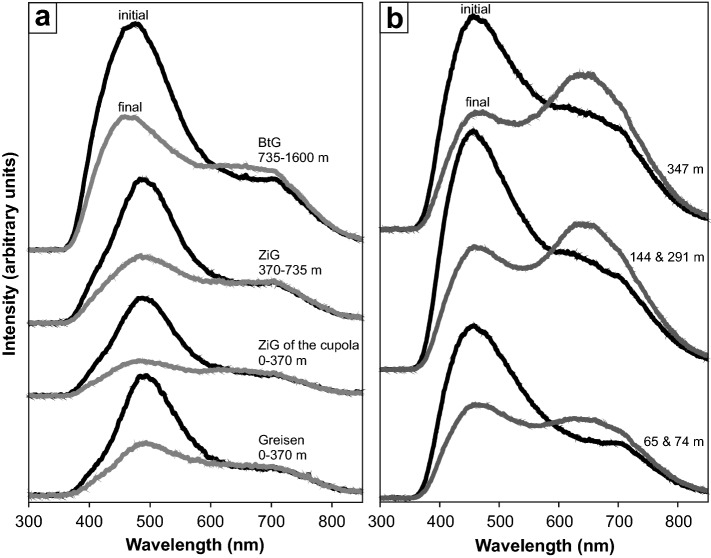
Average CL spectra from **a** Zinnwald and **b** Podlesí quartz at the start (black) and after two minutes (grey) of excitation. Changes in relative peak heights of the blue (around 500 nm, decreasing) and the red (around 700 nm, slightly increasing) portion of the spectra result in a shift towards more reddish CL colours. Grouping is based on the petrological classification of their host granites

In contrast to the diversity in the IR spectra, samples from both Zinnwald (Fig. 9a) and Podlesí (Fig. 9b) show rather uniform CL spectra. At the start of irradiation, the emission band at 500 nm dominates in all spectra, with subordinate portions of the emission bands at 390 nm and 420 nm in the range of blue visible light. In the region of red visible light, spectra are governed by several broad bands centred around 580 nm, 620 nm and 650 nm. A rather sharp emission band is located at 705 nm. After 2 min of electron irradiation, all spectra show a strong decline of the emission bands in the blue spectral region around 500 nm. While spectra from Podlesí quartz crystals exhibit a simultaneous increase in the red spectral region around 650 nm, Zinnwald quartz does not show a reaction to electron radiation in the same region.

### Trace element contents

**Fig. 10:**
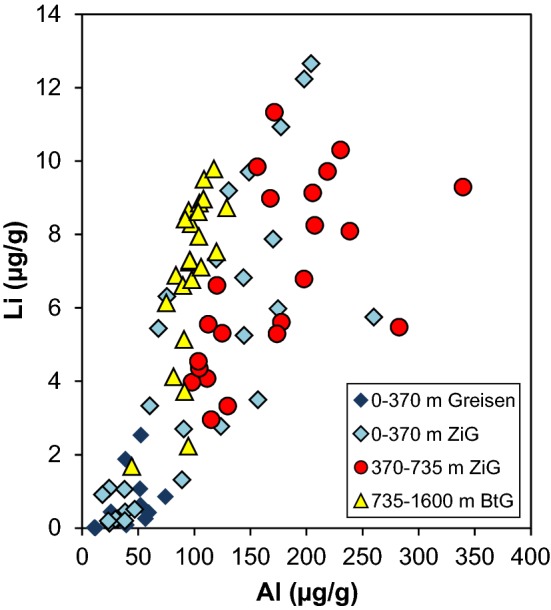
Li versus Al contents of Zinnwald quartz crystals from SIMS analyses. Values are grouped to display changes between different depth levels of the granitic body

Aluminium is the most abundant trace element in all analysed quartz crystals from Zinnwald (28–216 µg/g, Table 2), which is in accordance with previous studies (Götze et al. 2001). All other trace elements are 1–2 orders of magnitude lower. Since Al and Li are of most interest for comparison with the new IR data, illustrations focus on these two elements, but other elements such as B, Na and K exhibit nearly identical behaviour with depths. Between the surface and 735 m borehole depth, quartz crystals show an increase in almost all mono- and trivalent cations, mostly pronounced in the uppermost 370 m (Fig. 3c). Here, quartz crystals from greisen samples cluster systematically at lower Al and Li contents compared to crystals from granite samples. Below 735 m, all trace element contents drop, and then slightly increase towards 1600 m. Quartz crystals from the deepest level show a systematic higher Li/Al ratio compared to crystals from shallower depths (Fig. 10).

**Table 2:** Trace element concentrations in Zinnwald quartz

Sample #	Depth (m)	Crystal #	Li (µg/g)^a^	B (µg/g)^a^	Na (µg/g)^a^	Al (µg/g)^a^	K (µg/g)^a^	Ti (µg/g)^a^	Ge (µg/g)^a^	OH (µg/g)^b^
7	44	AP057	0.7 (0.6)	0.58 (0.08)	1.2 (0.2)	53 (31)	1.2 (0.3)	5.3 (0.6)	2.0 (0.5)	18.4
8	45	AP058	0.5 (0.3)	0.21 (0.02)	2.0 (0.2)	63 (10)	3.1 (0.8)	0.7 (0.0)	2.2 (0.5)	17.1
8	45	AP059	0.6 (0.5)	0.23 (0.11)	1.9 (0.2)	38 (23)	2.0 (0.6)	0.5 (0.1)	1.4 (0.9)	17.6
36	139	AP114	0.2 (0.2)	0.17 (0.10)	1.5 (0.2)	28 (13)	1.8 (0.2)	1.4 (2.7)	1.8 (1.2)	13.1
36	139	AP115	1.5 (1.2)	0.19 (0.12)	1.4 (0.1)	43 (8)	1.7 (0.3)	0.1 (0.0)	1.5 (0.2)	13.3
60	225	AP117	0.3 (0.2)	0.29 (0.02)	1.7 (0.3)	34 (12)	1.6 (0.3)	3.8 (0.3)	2.0 (0.2)	13.3
60	225	AP119	2.1 (1.7)	0.34 (0.13)	2.4 (0.5)	95 (60)	5.1 (2.5)	2.4 (0.9)	2.6 (0.5)	33.1
74	283	AP120	4.7 (1.7)	0.51 (0.20)	11.2 (7.0)	148 (26)	12.7 (7.6)	3.5 (0.2)	1.9 (0.3)	28.5
74	283	AP122	1.5 (1.6)	0.37 (0.17)	0.7 (0.7)	36 (21)	1.3 (1.1)	3.2 (0.2)	2.4 (0.2)	9.5
90	356	AP123	11.9 (0.9)	0.40 (0.06)	10.5 (2.1)	193 (14)	21.7 (4.3)	3.9 (0.0)	1.9 (0.3)	29.5
90	356	AP125	6.8 (4.0)	0.21 (0.06)	8.3 (4.3)	133 (59)	23.1 (11.3)	12.4 (8.4)	1.5 (0.6)	14.6
95	369	AP126	4.9 (3.5)	0.59(0.25)	6.2 (6.7)	156 (112)	11.1 (10.9)	4.1 (0.4)	2.1 (0.6)	30.1
95	369	AP127	6.2 (0.7)	0.23 (0.23)	7.0 (5.0)	96 (42)	11.0 (10.8)	26.0 (19.6)	1.1 (0.3)	11.1
131	540	AP076	10.0 (1.2)	0.61 (0.12)	12.1 (2.4)	165 (8)	25.4 (5.5)	3.3 (0.7)	2.0 (0.2)	15.0
131	540	AP077	6.3 (1.5)	1.40 (0.26)	21.4 (2.8)	197 (37)	51.4 (7.4)	4.3 (0.2)	2.5 (0.6)	20.0
160	733	AP078	8.9 (1.9)	0.47 (0.14)	19.9 (2.3)	216 (17)	41.3 (10.7)	3.4 (0.4)	2.3 (0.5)	23.7
160	733	AP133	5.3 (2.5)	1.23 (0.59)	30.4 (24.1)	199 (105)	57.0 (42.4)	4.1 (0.5)	2.4 (0.3)	25.8
170	797	AP081	7.4 (1.6)	0.44 (0.21)	5.2 (5.4)	162 (52)	16.2 (16.2)	6.2 (4.6)	2.0 (0.8)	26.6
170	797	AP136	4.2 (0.3)	0.22 (0.10)	0.8 (0.5)	105 (6)	2.0 (0.7)	16.1 (6.5)	1.1 (0.4)	25.1
198	1034	AP082	3.5 (1.7)	0.23 (0.12)	1.0 (0.4)	75 (27)	2.9 (1.5)	19.1 (10.7)	1.2 (0.5)	22.2
229	1340	AP085	8.1 (0.7)	0.14 (0.03)	0.5 (0.2)	96 (1)	1.2 (0.8)	18.3 (3.9)	1.0 (0.1)	16.0
229	1340	AP144	7.3 (1.0)	0.15 (0.01)	1.2 (0.3)	89 (4)	2.6 (0.6)	25.8 (16.6)	1.2 (0.2)	11.2
229	1340	AP145	7.3 (2.9)	0.31(0.19)	2.2 (1.8)	104 (14)	3.9 (2.9)	13.9 (4.3)	1.1 (0.2)	14.7
253	1578	AP086	8.2 (1.5)	0.21 (0.07)	1.2 (0.6)	106 (10)	3.0 (1.1)	17.1 (4.9)	1.2 (0.2)	22.7
253	1578	AP087	7.1 (0.5)	0.26 (0.11)	0.8 (0.6)	99 (16)	3.9 (4.0)	14.3 (6.7)	1.6 (1.0)	16.3

Values are averaged over 2–5 measurements per crystal and errors are given in brackets (1 sd)

^a^SIMS data

^b^FTIR data

Apart from the mono- and trivalent cations, the concentrations of tetravalent Ge do not show a discernible trend throughout the profile and remains close to the detection limit. Titanium contents, however, increase at depths of 735–1600 m, suggesting higher quartz crystallisation temperatures (Wark and Watson 2006; Huang and Audétat 2012).

## Discussion

### Incorporation mechanisms of impurities in quartz

**Fig. 11:**
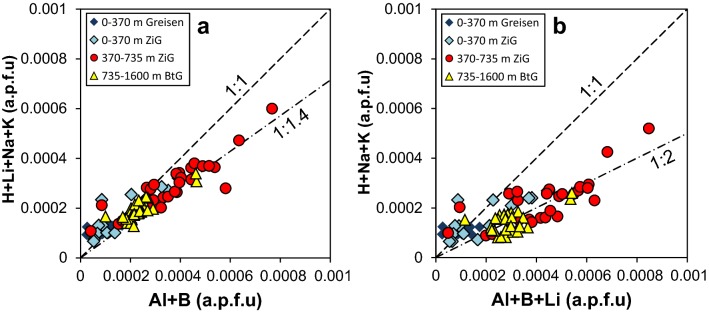
Atoms per formula unit (a.p.f.u.) plot of mono- versus trivalent cations. Endmember illustration of Lithium as charge balancer to form a **a** dry AlLi and **b** hydrous LiOH defect. Broken lines represent fixed ratios for orientation

Trace element contents deviate systematically from the 1:1 line (Fig. 11a, b) of the proposed charge balancing equation [H^+^] + [A^+^] + [P^5+^] = [M^3+^] + [B^3+^], with A^+^ = alkali ions and M^3+^ = metal ions (Müller and Koch-Müller 2009). The mismatch from the 1:1 line towards higher concentrations of trivalent cations indicates that there are additional incorporation mechanisms for the trivalent cations. One possible mechanism involves phosphorous by the substitution [Si^4+^] = [P^5+^] + [Al^3+^]. However, phosphorous was not included in the SIMS protocol for the reason that analyses of P require extensive modifications in the measurement strategy at the expense of the detection limits of other trace elements. On the other hand, the effect of P is probably small because of low P contents (< 10 µg/g) in Zinnwald quartz (Breiter et al. 2017b). As far as Li is concerned, data show a better correlation if Li is treated as H-free AlLi defect (Fig. 11a) than as a LiOH defect associated with non-bridging oxygens (Fig. 11b), which is in agreement with Frigo et al. (2016).

Total defect water contents mimic the variation in trace element content throughout the vertical section of the Zinnwald intrusion (Fig. 3). Since Al is the most abundant trace element in quartz, both FTIR spectra of quartz and their defect water contents are dominated by bands associated with AlOH defects, followed by LiOH defects. Hydrothermal greisen quartz from the most shallow part shows by far the lowest defect water content (Table 1; Fig. 3a), mirroring their low trace element contents (Table 2; Fig. 10). Quartz from granites (other than greisen) from the same depth exhibit a diverse pattern in both OH defect speciation and total defect water content, which is reflected by a large scatter in their trace element content (Fig. 10).

### Differentiation processes in plutonic bodies

The observed hydrous defect variations mirror changes in physico-chemical parameters during crystal growth, such as pressure, temperature, water activity and availability of trace metals. Zoned crystals (Fig. 8) or crystals that exhibit several generations (Fig. 7) thus reflect changes during the crystallisation at the same portion of the intrusion. Changes on a large scale reveal compositional zonations within plutonic bodies and give information about the differentiation history in the specific body. With respect to chemical variables, OH absorption bands point to changes in specific trace elements (e.g. Li, B) in the host rock. Physical variables such as pressure should in general also influence the defect chemistry. However, the suggested equilibration pressure for Zinnwald is around 1–2 kbar (Breiter et al. 2012) and the no overall trend of total defect water contents in quartz was observed in experiments between 1 and 3 kbar (Potrafke et al. 2019). Therefore, no clear pressure effect over the 1600-m-depth profile is expected. Similarly, for Podlesí Stock the increase in total defect water content with increasing depth over the narrow range of 350 m (Fig. 5a) cannot be explained by pressure. The reason for the trend either be (a) higher crystallisation temperatures due to greater distances from the cooler surrounding country rock, promoting OH incorporation, or (b) reduced water activity towards the uppermost part of the intrusion due to water loss. The latter is consistent with geological observations that document a strongly tourmalinised exocontact of the stock, explaining the contribution of BOH defects in quartz from the most shallow sample (Fig. 1b). The granite itself is tourmaline-free, indicating that nearly all B escaped with the fluid to the surrounding phyllites.

**Fig. 12:**
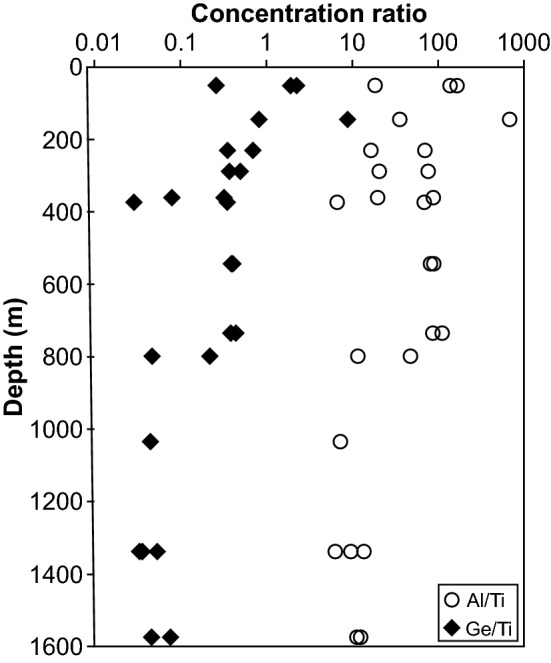
Molar trace element ratios of Al/Ti and Ge/Ti versus depths. Higher ratios indicate higher degrees of differentiation (Breiter et al. 2005; Müller et al. 2018)

The most important changes observed in the Zinnwald quartz sample set (Figs. 3a, 10) can be explained by changes in the lithology. The trace element content characteristics match the subdivision of the intrusive body in different depth levels as documented earlier (Štemprok and Šulcek 1969; Štemprok 2016; Breiter et al. 2017b), and is best reflected by the Al contents. In the uppermost 370 m, quartz of hydrothermal origin (“greisen stage”) can be distinguished from quartz from the ZiG (Zinnwaldite granite) by their low overall trace element contents (Table 2; Breiter et al. 2015). This depth level represents the most fractionated part of the intrusion and promotes the incorporation of incompatible elements in quartz. Highest Al contents are reached in ZiG quartz between 370 and 735 m. The ZiG–BtG transition at around 735 m can be easily identified by OH defects in terms of a drop in total defect water content, mainly caused by a drop in Al content. In general, hydrous defects in all quartz from Zinnwald match the petrological classification of the body (Greisen–ZiG–BtG). Values for Al/Ti and Ge/Ti increase noticeably from the ZiG–BtG transition upwards (Fig. 12), which was interpreted as a higher degree of fractionation for ZiG that leads to a maximum in greisen quartz due to their low Ti contents (Breiter et al. 2017a).

In contrast to the Zinnwald intrusion, the discrimination of quartz from the Podlesí sample set is more complex, since six quartz generations (PQ1–6) from two different intrusion stages, i.e. different age and magma chemistry, were identified earlier (Breiter and Müller 2009). However, only samples from the first intrusion stage (‘stock granite’, PQ1–2) were considered, and quartz from a second intrusion stage as well as late hydrothermal quartz (PQ3–6) is not included in the present data set. In the investigated granite facies, only sub-euhedral quartz phenocrysts (PQ1) and fine-grained groundmass quartz (PQ2) occur, with a greater portion of PQ1. Therefore, results for Podlesí crystals only reflect possible differentiation trends within one quartz generation.

### Tool for provenance analyses of eroded material

Over geological time scales, plutonic bodies are uplifted, eroded and finally transported into sedimentary systems. In a sedimentary sequence, older (lower) strata represent the upper regions of eroded plutons and younger (upper) strata tend to represent the lower levels of the igneous body. Monitoring and understanding of the distribution of defect water in plutonic bodies thus helps to define grain clusters that may be identified in sediments that (partly) are supplied by this igneous body. The application of OH defects in quartz as a tool for provenance analyses of river sediments (Stalder et al. 2017, 2019; Jaeger et al. 2019) would give more conclusive results, if systematic data on the largest intrusion in the catchment area were available. Furthermore, quartz from sediments may be of particular interest for industrial resource prospection, e.g. strong LiOH absorption bands may (though do not necessarily have to) indicate a potential Li deposit in the catchment area.

**Fig. 13:**
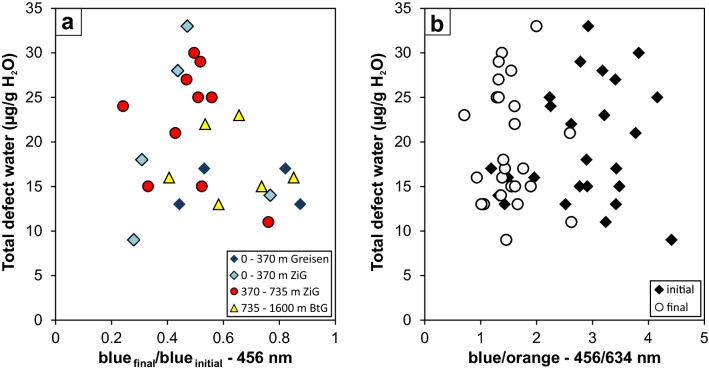
**a** Correlation of total defect water content with CL band ratios in the blue colour range (centred at ~ 456 nm) prior to and after irradiation. **b** Correlation of total defect water content with the blue to orange (centred at 634 nm) ratio, prior to and after excitation

In agreement with previously published results on random quartz crystals from sedimentary material (Stalder and Neuser 2013), there is no apparent correlation between the defect water content and CL band characteristics (Fig. 13). Yet, electron irradiation (during CL analysis) changes the intensity of the dominant blue emission line (Fig. 13a), but without correlation of the total defect water content to any group of samples. The same holds true for the ratio between blue (centred at ~ 456 nm) and red (centred at ~ 634 nm) CL colours (Fig. 13b). In general, IR spectra are easier to interpret because their bands are sharper and exhibit less overlap. Both methods (IR and CL) need a comparable effort in terms of preparation, analysis time and costs, but reveal complementary information: CL spectroscopy provides reliable results for discrimination of quartz of different origins (volcanic, plutonic, metamorphic and authigenic) mainly based on the cocktail of incorporated trace metals. In contrast, IR spectra are sensitive indicators for subtle changes of volatile components in the igneous system, or—alternatively—indicate whether defect water was destroyed by metamorphic overprint. A comparison of both methods is not always straightforward, because of the different amount of probed sample volumes in lateral and vertical dimensions. CL images the surface, while FTIR measures the absorption through the whole thickness of the crystal. Changes on the small scale will only be recorded if they are monotonous such as in diffusion or growth profiles.

## Conclusions

The hydrous defect chemistry of igneous quartz mimics the variation in trace element contents and shows characteristic variations that reflect changes in the formation condition such as the availability of trace metals and water. The defect chemistry throughout a vertical section of a plutonic body exhibits trends and arrays that are typical for (but unique to) the respective intrusion and documents changes in lithology. Defined arrays of OH absorption characteristics measured by IR spectroscopy can thus be used as tool for provenance analyses of the eroded material. IR spectra are more sensitive to slight changes in defect inventory compared to CL spectra.

## Electronic supplementary material

Below is the link to the electronic supplementary material.

Supplementary material 1 (PDF 103 kb)

## References

[CR1] Ackerman L, Haluzová E, Creaser RA, Pašava J, Veselovský F, Breiter K, Erban V, Drábek M (2017). Temporal evolution of mineralization events in the Bohemian Massif inferred from the Re–Os geochronology of molybdenite. Miner Depos.

[CR2] Ackerson MR, Tailby ND, Watson EB (2015). Trace elements in quartz shed light on sediment provenance. Geochem Geophys.

[CR3] Aines RD, Rossman GR (1984). Water in minerals? A peak in the infrared. J Geophys Res.

[CR4] Augustsson C, Reker A (2012). Cathodolumenescence spectra of quartz as provenance indicators revisited. J Sediment Res.

[CR5] Bambauer HU (1961). Spurenelementgehalte und γ-Farbzentren in Quarzen aus Zerrklüften der Schweizer Alpen. Schweiz Mineral Petrogr Mitt.

[CR6] Bambauer HU (1963). Merkmale des OH-Spektrums alpiner Quarze (3μ-Gebiet). Schweiz Mineral Petrogr Mitt.

[CR7] Baron MA, Stalder R, Konzett J, Hauzenberger CA (2015). OH-point defects in quartz in B- and Li-bearing systems and their application to pegmatites. Phys Chem Miner.

[CR8] Bottazzi P, Ottolini L, Vannucci R (1992). SIMS analyses of rare earth elements in natural minerals and glasses: an investigation of structural matrix effects on ion yields. Scanning.

[CR9] Breiter K (2012). Nearly contemporaneous evolution of the A- and S-type fractionated granites in the Krušné hory/Erzgebirge Mts., Central Europe. Lithos.

[CR10] Breiter K, Müller A (2009). Evolution of rare-metal granitic magmas documented by quartz chemistry. Eur J Mineral.

[CR11] Breiter K, Müller A, Leichmann J, Gabašová A (2005). Textural and chemical evolution of a fractionated granitic system: the Podlesí stock, Czech Republic. Lithos.

[CR12] Breiter K, Svojtka M, Ackerman L, Švecová K (2012). Trace element composition of quartz from the Variscan Altenberg-Teplice caldera (Krušné hory/Erzgebirge Mts, Czech Republic/Germany): insights into the volcano-plutonic complex evolution. Chem Geol.

[CR13] Breiter K, Ďurišová J, Korbelová Z, Vaňková M, Vašinová M, Breiter K (2015). Quartz, micas and ore minerals as expression of vertical zoning in the drill hole CS-1. Workshop on the vertical zoning of ore-bearing granite plutons in light of modern analytical methods, October 6, 2015.

[CR14] Breiter K, Ďurišová J, Dosbaba M (2017). Quartz chemistry—a step to understanding magmatic-hydrothermal processes in ore-bearing granites: Cínovec/Zinnwald Sn–W–Li deposit, Central Europe. Ore Geol Rev.

[CR15] Breiter K, Ďurišová J, Hrstka T, Korbelová Z, Hložková Vaňková M, Vašinová Galiová M, Kanický V, Rambousek P, Knésl I, Dobeš P, Dosbaba M (2017). Assessment of magmatic vs. metasomatic processes in rare-metal granites: a case study of the Cínovec/Zinnwald Sn–W–Li deposit, Central Europe. Lithos.

[CR16] Cháb J, Breiter K, Fatka O, Hladil J, Kalvoda J, Šimůnek Z, Štorch P, Vašíček Z, Zajíc J, Zapletal J (2010). Outline of the geology of the Bohemian Massif.

[CR17] Currie LA (1968). Limits for qualitative detection and quantitative determination. Anal Chem.

[CR18] Förster H-J, Romer RL, Linnemann U, Romer RL (2010). Carboniferous magmatism. Pre-Mesozoic geology of Saxo-Thuringia—from the Cadomian active margin to the Variscan orogen.

[CR19] Frigo C, Stalder R, Hauzenberger CA (2016). OH defects in quartz in granitic systems doped with spodumene, tourmaline and/or apatite: experimental investigations at 5–20 kbar. Phys Chem Miner.

[CR20] Gorton NT, Walker G, Burley SD (1997). Experimental analysis of the composite blue CL emission in quartz. J Lumin.

[CR21] Götze J, Plötze M, Habermann D (2001). Origin, spectral characteristics and practical applications of the cathodoluminescence (CL) of quartz—a review. Mineral Petrol.

[CR22] Holtz F, Johannes W (1994). Maximum and minimum water contents of granitic melts: implications for chemical and physical properties of ascending magmas. Lithos.

[CR23] Hoth K, Wasternack J, Berger H-J, Breiter K, Mlčoch B, Schovánek P (1995). Geologische Karte Erzgebirge-Vogtland 1:100 000.

[CR24] Huang R, Audétat A (2012). The titanium-in-quartz (TitaniQ) thermobarometer: a critical examination and re-calibration. Geochim Cosmochim Acta.

[CR25] Jaeger D, Stalder R, Masago H, Strasser M (2019). OH defects in quartz as a provenance tool: application to fluvial and deep marine sediments from SW Japan. Sediment Geol.

[CR26] Jochum KP, Weis U, Stoll B, Kuzmin D, Yang Q, Raczek I, Jacod DE, Stracke A, Birbaum K, Frick DA, Günther D, Enzweiler J (2011). Determination of reference values for NIST SRM 610–617 glasses following ISO guidelines. Geostand Geoanal Res.

[CR27] Kaliwoda M, Marschall HR, Marks MAW, Ludwig T, Altherr R, Markl G (2011). Boron and boron isotope systematics in the peralkaline Ilímaussaq intrusion (South Greenland) and its granitic country rocks: a record of magmatic and hydrothermal processes. Lithos.

[CR28] Kats A (1962). Hydrogen in alpha quartz. Philips Res Rep.

[CR29] Kleine BI, Stefánsson A, Halldórsson SA, Whitehouse MJ, Jónasson K (2018). Silicon and oxygen isotopes unravel quartz formation processes in the Icelandic crust. Geochem Perspect Lett.

[CR30] Larsen RB, Henderson I, Ihlen PM, Jacamon F (2004). Distribution and petrogenetic behaviour of trace elements in granitic pegmatite quartz from South Norway. Contrib Mineral Petrol.

[CR31] Libowitzky E, Rossman GR (1997). An IR calibration for water in minerals. Am Mineral.

[CR32] Linnemann U, Romer RL (2010). Pre-Mesozoic geology of Saxo-Thuringia—from the Cadomian active margin to the Variscan orogen.

[CR33] Luff BJ, Townsend PD (1990). Cathodoluminescence of synthetic quartz. J Phys Condens Matter.

[CR34] MacRae ND, Bottazzi P, Ottolini L, Vannucci R (1993). Quantitative REE analysis of silicates by SIMS: conventional energy filtering vs. specimen isolation mode. Chem Geol.

[CR35] Marschall HR, Ludwig T (2004). The low-boron contest: minimising surface contamination and analysing boron concentrations at the ng/g-level by secondary ion mass spectrometry. Mineral Petrol.

[CR36] Müller A, Koch-Müller M (2009). Hydrogen speciation and trace element contents of igneous, hydrothermal and metamorphic quartz from Norway. Mineral Mag.

[CR37] Müller A, Seltmann R, Behr HJ (2000). Application of cathodoluminescence to magmatic quartz in a tin granite—case study from the Schellerhau Granite Complex, Eastern Erzgebirge, Germany. Miner Depos.

[CR38] Müller A, René M, Behr HJ, Kronz A (2003). Trace elements and cathodoluminescence of igneous quartz in topaz granites from the Hub Stock (Slavkovský Les Mts., Czech Republic). Mineral Petrol.

[CR39] Müller A, Wiedenbeck M, Van den Kerkhof AM, Kronz A, Simon K (2003). Trace elements in quartz—a combined electron microprobe, secondary ion mass spectrometry, laser-ablation ICP-MS, and cathodoluminescence study. Eur J Mineral.

[CR40] Müller A, Herklotz G, Giegling H (2018). Chemistry of quartz related to the Zinnwald/Cínovec Sn–W–Li greisen-type deposit, Eastern Erzgebirge, Germany. J Geochem Explor.

[CR41] Neuser RD, Bruhn F, Götze J, Habermann D, Richter DK (1996). Kathodolumineszenz: Methodik und Anwendung. Zbl Geol Paläont.

[CR42] Ottolini L, Bottazzi P, Vannucci R (1993). Quantification of lithium, beryllium, and boron in silicates by secondary ion mass spectrometry using conventional energy filtering. Anal Chem.

[CR43] Ottolini L, Cámara F, Hawthorne FC, Stirling J (2002). SIMS matrix effects in the analysis of light elements in silicate minerals: comparison with SREF and EMPA data. Am Mineral.

[CR44] Perny B, Eberhardt P, Ramseyer K, Mullis J, Pankrath R (1992). Microdistribution of Al, Li, and Na in α-quartz: possible causes and correlation with short-lived cathodoluminescence. Am Mineral.

[CR45] Potrafke A, Stalder R, Schmidt BC, Ludwig T (2019). OH defect contents in quartz in a granitic system at 1–5 kbar. Contrib Mineral Petrol.

[CR46] Ramseyer K, Mullis J (1990). Factors influencing short-lived blue cathodoluminescence of alpha-quartz. Am Mineral.

[CR47] Rovetta MR (1989). Experimental and spectroscopic constraints on the solubility of hydroxyl in quartz. Phys Earth Planet Int.

[CR48] Seltmann R, Schilka W (1995). Late-Variscan crustal evolution in the Altenberg-Teplice caldera. Evidence from new geochemical and geochronological data. Terra Nostra.

[CR49] Shimizu N (1986). Silicon-induced Enhancement in secondary ion emission from silicates. Int J Mass Spec Ion Process.

[CR50] Stalder R (2014). OH-defect content in detrital quartz grains as an archive for crystallisation conditions. Sediment Geol.

[CR51] Stalder R, Konzett J (2012). OH-defects in quartz in the system quartz–albite–water and granite water between 5 and 25 kbar. Phys Chem Miner.

[CR52] Stalder R, Neuser RD (2013). OH-defects in detrital quartz grains: potential for application as tool for provenance analysis and overview over crustal average. Sediment Geol.

[CR53] Stalder R, Potrafke A, Billström K, Skogby H, Meinhold G, Gögele C, Berberich T (2017). OH defects in quartz as monitor for igneous, metamorphic, and sedimentary processes. Am Mineral.

[CR54] Stalder R, von Eynatten H, Costamoling J, Potrafke A, Dunkl I, Meinhold G (2019). OH in detrital quartz grains as tool for provenance analysis: case studies on various settings from Cambrian to Recent. Sediment Geol.

[CR55] Štemprok M (2016). Drill hole CS-1 penetrating the Cínovec/Zinnwald granite cupola (Czech Republic): an A-type granite with important hydrothermal mineralization. J Geosci.

[CR56] Štemprok M, Šulcek Z (1969). Geochemical profile through an ore-bearing lithium granite. Econ Geol.

[CR57] Stevens-Kalceff MA (2009). Cathodoluminescence microcharacterization of point defects in α-quartz. Mineral Mag.

[CR58] Stevens-Kalceff MA, Phillips MR (1995). Cathodoluminescence microcharacterization of the defect structure of quartz. Phys Rev B.

[CR59] Thomas SM, Koch-Müller M, Reichart P, Rhede D, Thomas R, Wirth R (2009). IR calibrations for water determination in olivine, r-GeO_2_ and SiO_2_ polymorphs. Phys Chem Miner.

[CR60] Tuttle OF, Bowen NL (1958) Origin of granite in the light of experimental studies in the system NaAlSi_3_O_8_–KAlSi_3_O_8_–SiO_2_–H_2_O. Geol Soc Am Mem 74

[CR61] Wark DA, Watson EB (2006). TitaniQ: a titanium-in-quartz geothermometer. Contrib Mineral Petrol.

[CR62] Watt GR, Wright P, Galloway S, McLean C (1997). Cathodoluminescence and trace element zoning in quartz phenocrysts and xenocrysts. Geochim Cosmochim Acta.

[CR63] Zinkernagel U (1978). Cathodoluminescence of quartz and its application to sandstone petrology. Contrib Sedimentol.

[CR64] Zinner E (1986). A method for the quantitative measurement of rare earth elements in the ion microprobe. Int J Mass Spectrom Ion Process.

