# Supplementary material for: Variations of OH defects and chemical impurities in natural quartz within igneous bodies
Source: Phys Chem Miner. 2020 May 5;47(5):24. doi: 10.1007/s00269-020-01091-w (PMC7200648; doi:10.1007/s00269-020-01091-w)
Supplement: Supplementary file 1 — Supplementary material 1 (PDF 103 kb) [file 269_2020_1091_MOESM1_ESM.pdf]

Electronic appendix Potrafke et al. OH defects in quartz: application to igneous bodies from the Erzgebirge
Details on SIMS analyses: Calibration, background and detection limits.

RIY calibration analyses on NIST SRM 610 glass

	RIY Li/Si	RIY B/Si	RIY Na/Si	RIY Al/Si	RIY K/Si	RIY Ti/Si	RIY Ge/Si
SRM610-1	1.465	0.543	0.925	2.381	1.104	4.296	0.218
SRM610-2	1.455	0.544	0.925	2.390	1.092	4.272	0.216
SRM610-3	1.476	0.544	0.924	2.382	1.089	4.308	0.215
SRM610-4	1.483	0.546	0.927	2.385	1.105	4.251	0.211
SRM610-5	1.467	0.541	0.931	2.389	1.105	4.321	0.208
SRM610-6	1.477	0.544	0.933	2.402	1.092	4.328	0.214
SRM610-7	1.477	0.542	0.928	2.392	1.103	4.341	0.212
Mean RIY	1.471	0.543	0.928	2.389	1.099	4.302	0.213
1sd	0.010	0.002	0.003	0.007	0.007	0.032	0.003
1rsd	0.7%	0.3%	0.4%	0.3%	0.7%	0.7%	1.6%

Secondary ion intensities (counts/s) on NIST SRM 610 glass

	^{7}Li	^{11}B	^{23}Na	^{27}Al	^{39}K	^{48}Ti	^{74}Ge
SRM610-1	7.58E+03	1.17E+03	3.32E+05	7.56E+04	1.01E+03	2.48E+03	4.03E+01
SRM610-2	7.95E+03	1.24E+03	3.50E+05	8.01E+04	1.06E+03	2.61E+03	4.23E+01
SRM610-3	8.00E+03	1.23E+03	3.47E+05	7.92E+04	1.05E+03	2.61E+03	4.18E+01
SRM610-4	8.01E+03	1.23E+03	3.47E+05	7.90E+04	1.06E+03	2.56E+03	4.09E+01
SRM610-5	8.03E+03	1.23E+03	3.53E+05	8.02E+04	1.07E+03	2.64E+03	4.07E+01
SRM610-6	8.00E+03	1.23E+03	3.51E+05	7.98E+04	1.05E+03	2.62E+03	4.15E+01
SRM610-7	7.92E+03	1.21E+03	3.45E+05	7.87E+04	1.05E+03	2.60E+03	4.07E+01
Mean	7.93E+03	1.22E+03	3.47E+05	7.89E+04	1.05E+03	2.59E+03	4.12E+01

Concentrations in NIST SRM 610 glass (Jochum et al. 2011)

	Li	B	Na	Al	K	Ti	Ge
**c (µg/g)**	468	350	99409	10320	464	452	447

Background and detection limit (see Marschall & Ludwig 2004 and Currie 1968)

	Li	B	Na	Al	K	Ti	Ge
S_{abs} (counts s^{-1} (µg/g)$^{-1}$)	16.941	3.478	3.486	7.650	2.264	5.725	0.092
Total integration time (s)	32	64	32	32	32	64	128
"Well-known" background (counts/s)	0.025	0.025	0.025	0.025	0.025	0.025	0.025
μ_b (counts)	0.800	1.600	0.800	0.800	0.800	1.600	3.200
S_C (counts)	1.467	2.074	1.467	1.467	1.467	2.074	2.934
S_D (counts)	5.653	6.872	5.653	5.653	5.653	6.872	8.595
"Well-known" background (µg/g)	0.001	0.007	0.007	0.003	0.011	0.004	0.271
x_C (µg/g)	0.003	0.009	0.013	0.006	0.020	0.006	0.249
x_D (µg/g)	0.010	0.031	0.051	0.023	0.078	0.019	0.729

Test analyses on silica glass Herasil 102 (conc. in µg/g)

	Li	B	Na	Al	K	Ti	Ge
Herasil 102-1	0.087	0.013	0.126	7.506	0.307	0.047	-0.124
Herasil 102-2	0.091	0.007	0.184	8.063	0.249	0.021	0.256
Herasil 102-3	0.070	0.010	0.235	8.839	0.399	0.049	-0.129
Mean	0.083	0.010	0.182	8.136	0.319	0.039	0.001
1sd	0.011	0.003	0.055	0.669	0.076	0.016	0.221
